# Two rounds of the Pandemic Fund at the WHO Eastern Mediterranean Region: progress, lessons learned, challenges, and way forward

**DOI:** 10.1080/16549716.2025.2475596

**Published:** 2025-05-09

**Authors:** Mohamed Elhakim, Amgad Elkholy, Mary Stephen, Richard John Brennan, Ahmed Zouiten

**Affiliations:** a Health Security and Preparedness, WHO Health Emergencies Program, World Health Organization Eastern Mediterranean Regional Office, Cairo, Egypt; b Regional Emergency Director, WHO Health Emergencies Program, World Health Organization Eastern Mediterranean Regional Office, Cairo, Egypt; c Regional Emergency Director (a.i.), WHO Health Emergencies Department, World Health Organization Eastern Mediterranean Regional Office, Cairo, Egypt/WHO Representative Libya Country Office, Tripoli, Libya

**Keywords:** Pandemic Fund, Eastern Mediterranean Region, global health security, International Health Regulations, health preparedness, COVID-19 gains

## Abstract

World Health Organization (WHO) played an important role in supporting the Member States of the Eastern Mediterranean Region (EMR) apply for awards from the Pandemic Fund – a vital source of funding for pandemic preparedness and response. The aim of this commentary is to reflect the contributions of WHO EMR during the first two funding rounds of the Pandemic Fund, while highlighting the lessons learned, and addressing the ongoing challenges faced across the region. As EMR Member States continue to build and strengthen their health security capacities, WHO’s involvement has catalyzed the improvement of early warning systems, laboratory capacity, and workforce development. However, challenges remain, including sustainability, enhancing regional cooperation, the widespread state fragility, and multiple conflicts across the region.

## Paper Context

- **Main findings**: The paper highlights significant improvements in proposal development and funding allocation in the second round of the Pandemic Fund for the Eastern Mediterranean Region, demonstrating the World Health Organization’s important role in enhancing regional pandemic preparedness as the main Implementing Entity.
- **Added knowledge**: It provides insights into the lessons learned, including the importance of regional collaboration, sustainability, and equity in resource mobilization and distribution, offering practical recommendations for future funding and preparedness efforts in the Eastern Mediterranean Region.
- **Global health impact for policy and action**: The findings underline the necessity of integrating pandemic preparedness into national health systems and adopting cross-border activities to enhance health resilience and global health security.

## Background

Established in September 2022 by the World Bank in collaboration with the World Health Organization as technical lead, the Pandemic Fund, supported by the G20, addresses essential gaps in global pandemic preparedness and response (PPR) through targeted financial support to low- and middle-income countries. It aims to catalyze additional financial resources, encouraging countries to increase their own investments in pandemic preparedness. The Pandemic Fund enhances coordination among partners and serves as a platform for advocacy, to ensure sustained high-level recognition to strengthen health security. WHO serves as a technical lead and Implementing Entity (IE) by supporting the development, implementation, and monitoring of funded activities and provides expertise to ensure alignment with global health standards [1]. WHO Eastern Mediterranean Regional Office (EMRO) played an essential role in guiding eligible countries within the region through the process by providing technical and implementation support, reporting on progress and ensuring alignment with global health preparedness standards. These responsibilities, of the IE, ensure that pandemic preparedness investments are effectively implemented, monitored, and sustained.

Nine out of the twenty-two Member States of the Eastern Mediterranean Region (EMR) are classified as fragile and conflict-affected situations by the World Bank [2]. The health systems in these countries face significant challenges in delivering essential health services due to ongoing political instability, weakened infrastructure, and limited resources. They struggle with disruptions in health governance, leading to fragmented service delivery, poor access to health care, and overburdened healthcare facilities. The lack of security exacerbates shortages of medical supplies, trained personnel, and proper health data collection and information sharing, which interferes with timely responses to disease outbreaks [3,4]. Despite these challenges, efforts by WHO EMRO and partners are focusing on strengthening Member States resilience through targeted interventions, including resource mobilization to rebuild the needed capacities for preparedness to the next pandemic.

## Development of the Pandemic Fund proposals in EMR

During the Pandemic Fund proposal development process, WHO, as the IE of the fund, worked closely with national concerned ministries, including the health, agriculture, and finance, to assess the gaps in pandemic preparedness and align the proposals with the Pandemic Fund’s three key priority areas: early warning and disease surveillance systems, laboratory capacity, and strengthening the health workforce. WHO EMRO provided active support to Member States in developing the Pandemic Fund proposals through technical assistance, coordination, and capacity building, with close follow-up by WHO Country Offices.

The first round of funding, completed in July 2023, awarded 338 million USD globally, with two EMR countries benefiting: Palestine ($20,000,000) and Yemen ($26,020,000) [5]. The second round, announced on October 2024, provided an additional 547 million USD globally, including 128.89 million USD specifically fast-tracked for the mpox response in Africa. Across the EMR, there were substantial improvements in proposal quality and strategic planning among the region’s eligible countries, with five countries successfully receiving awards – Egypt ($23,054,221), Jordan ($4,024,530), Lebanon ($11,440,000), Pakistan ($18,675,609.63), and Tunisia ($25,000,000). Three additional Member States from the EMR, Djibouti, Somalia, and Sudan, benefitted from the regional mpox fast-tracked proposal, known as Preparedness for Pandemic Response (PREPARE), which was awarded a total of $31,934,205. This regional initiative extended beyond the EMR, incorporating four additional Member States from the WHO African Region, including, Ethiopia, Kenya, South Sudan, and Uganda. The PREPARE proposal was designed to strengthen multi-country pandemic preparedness efforts by leveraging a regional approach to surveillance, early detection, and response coordination [6]. The Intergovernmental Authority on Development (IGAD) played a crucial role as the regional coordinating entity, facilitating collaboration among the participating countries, with WHO as the IE of the proposal. Additionally, the International Federation of Red Cross and Red Crescent Societies (IFRC) was designated as the delivery partner, responsible for implementing key preparedness activities and ensuring operational support at the national and regional levels. This approach enabled resource-sharing, enhanced cross-border coordination, and addressed pandemic threats that extend beyond individual country borders.

If the two rounds of the Pandemic Fund are compared in EMR, significant improvements were seen in the second round of funding, which was awarded on 18 October 2024. WHO EMRO was continuously engaged through online sessions, technical guidance, and proposal reviews, which helped the 13 eligible countries refining their applications and address the shortcomings identified during the first round. Hence, the second round saw more comprehensive and aligned proposals, with five single country proposals and one regional proposal accepted, securing funds for eight eligible Member States from the region [6,7].

From the experience of the first round, the need for stronger regional and multi-country collaboration was obvious. WHO EMRO encouraged countries to participate in multi-country or regional proposals to leverage shared resources and address cross-border health threats. This approach increased the possibility of securing funding while also promoting cooperation among neighboring countries, which is essential for pandemic preparedness in a region with significant armed conflicts, political, and economic instability.

## Lessons learned

From the first and second rounds of the Pandemic Fund, many lessons were learned to improve the coordination and the quality of the submitted proposals including:

- **Strengthened proposal development:** The first round of funding highlighted the need for high-level technical assistance in proposal writing. WHO EMRO provided support to countries, including online sessions on developing coherent and evidence-based proposals, and encouraged Member States to hire dedicated technical experts for this purpose. This approach led to a higher success rate in the second round, with countries submitting technically stronger, more detailed proposals that addressed both national and regional PPR gaps.
- **Financial sustainability:** One of the lessons from both rounds is the importance of financial sustainability. While funding helps countries build early warning systems and laboratory capacities, the question remains, how will these gains be sustained once Pandemic Fund ends? WHO EMRO started accelerating the need for co-financing/co-investment and integrating PPR into national health budgets, ensuring that improvements are not dependent solely on external funding.
- **Equity in resource distribution:** One of the main challenges of the Pandemic Fund is to ensure equitable distribution of resources. Several EMR countries, particularly those directly affected by conflicts, face significant health system challenges affecting their capacity to develop competitive proposals. WHO EMRO worked to provide additional support to these countries to ensure that they are not left behind in both rounds of funding, and the results of the first round with Palestine and Yemen benefiting from the fund is the best example.
- **Sustain COVID-19 gains:** A key message from the COVID-19 pandemic is the importance of building on and sustaining the gains achieved during the global response. During the pandemic, EMR countries, substantially scaled up their PPR capacities, including improvements in surveillance systems, laboratory infrastructure, and workforce development. However, few countries had clear plans to sustain these gains post-COVID-19. WHO EMRO has emphasized the integration of these gains into Pandemic Fund proposals. A specific session was organized, in April 2024, to explain to all eligible countries focal points the necessity of institutionalizing the COVID-19 gains built within countries to ensure that they are resilient and adaptable for future health emergencies and crises.

## Challenges

Despite the improvements in quality and technical content of the EMR proposals during the second round, many challenges remain as obstacles for the Pandemic Fund implementation in the EMR. The first is the political instability in several Member States, which affects both the implementation of PPR activities and the ability to secure future funding. Countries like Palestine [8], Somalia [9], Sudan [10], and Yemen [11] face immense difficulties in maintaining basic health services, with huge negative impact on preparedness for future pandemics.

Another challenge is the regional and multi-country cooperation. While, WHO EMRO has successfully promoted regional and multi-country proposals, the cross-border surveillance and laboratory networks remain underdeveloped and even subject to attacks, due to the ongoing war in Gaza [12] and other conflicts. Political tensions and poor infrastructure have disrupted the creation of resilient regional health systems at the points of entry, a key component of effective pandemic prevention, preparedness, and response.

Finally, funds sustainability remains a major concern in EMR. As the second round of Pandemic Fund grants should start to be implemented soon, countries have to ensure that investments in pandemic preparedness are integrated into broader health system strengthening efforts. WHO EMRO is advocating for the governments to commit to long-term funding for PPR activities to avoid dependence on external grants.

## Way forward – implementation phase

As stated in the guidance note for applicants on the second call for proposals of the Pandemic Fund, the IE, according to its scope and mandate, has five major roles to play including: administrating the financial intermediary fund (FIF) transferred to it; conducting discussions with beneficiary countries on activities mentioned in the proposal; providing implementation support to the beneficiary countries; providing financial and progress report to the Governing Board of the Pandemic Fund through both the Trustee and the Secretariat; and cooperating on reviews and evaluations of the FIF under terms acceptable to the IE [13].

Looking forward, the real success of the Pandemic Fund opportunity depends on effective implementation at both national level, for single country proposals, and the regional level, for the regional proposal. WHO, as the IE, will continue to provide technical support, ensuring that projects are aligned with global standards for pandemic preparedness, and that PPR activities are sustained beyond the initial funding cycles [14]. Furthermore, the vast experience and extensive field presence of WHO will allow the real-time operational support, to EMR Member States, during the implementation phase, which includes monitoring and evaluation of different projects. Finally, WHO will continue playing the role of facilitating partnerships between governments, non-governmental organizations, civil society organizations, and multilateral development banks, which is essential to mobilize further resources and address gaps that emerge during implementation.

## Conclusion

The Pandemic Fund is a very useful opportunity for advancing pandemic preparedness across the WHO EMR. Through WHO EMRO’s technical support and strategic guidance, countries have made significant improvements in the proposals submitted to strengthen their health security level. However, many challenges remain in EMR, particularly around political instability, regional cooperation, and sustainability. As we move forward for the implementation, WHO EMRO will continue to play a key role in ensuring that pandemic preparedness is integrated into national health strategies, securing a safer future for the region’s populations’ health.
